# Human ex vivo comparison of *Escherichia coli* and *Pseudomonas aeruginosa* lipopolysaccharide–induced immune responses and cefiderocol effects in whole blood

**DOI:** 10.1007/s00430-026-00883-1

**Published:** 2026-07-27

**Authors:** Lena Pracher, Anselm Jorda, Sabine Eberl, Maria Weber, Markus Zeitlinger

**Affiliations:** Department of Clinical Pharmacology, Medical University of Vienna, Waehringer Guertel 18-20, 1090 Vienna, Austria

**Keywords:** Sepsis, Immune system, Cytokine, Antibiotic

## Abstract

Immune dysregulation is central in bacterial sepsis with lipopolysaccharide (LPS) of gram-negative bacteria acting as a key component in innate immune activation. Modulation of the host response by potential immunomodulatory agents may confer benefits. This ex vivo study compared cytokine responses induced by *E. coli* and *P. aeruginosa* LPS and evaluated the impact of cefiderocol on LPS-induced immune activation in human whole blood. Whole blood from ten healthy volunteers was stimulated ex vivo with *E. coli* or *P. aeruginosa* LPS in the presence or absence of cefiderocol and incubated for 4 h at 37 °C. Plasma concentrations of TNF-α, IL-1β, IL-6, IL-10, IFN-α, IFN-γ, and IL-8 were quantified at 0 h, 2 h, and 4 h using a multiplex immunoassay. Cytokine release was significantly higher after *E. coli* LPS stimulation than with *P. aeruginosa* LPS across all analytes. Mean TNF-α area under the curve (AUC₀–₄) was 2379 pg*h/mL with cefiderocol vs 1936 pg*h/mL without (percent difference 18.2% [95% CI, 5.9–31.4]) after *E. coli* LPS stimulation, and 278 pg/mL vs 242 pg*h/mL (12.9% [4.3–21.5]) after *P. aeruginosa* LPS stimulation. For IL-6, cefiderocol slightly increased AUC₀–₄ following *E. coli* LPS stimulation (4584 vs 3635 pg*h/mL; 20.7% [8.3–33.0]) but not with *P. aeruginosa* LPS (184 vs 201 pg*h/mL; − 9.3% [− 53.7–35.2]). IL-1β, IL-10, IFN-α, IFN-γ, and IL-8 levels were mostly unaffected by cefiderocol. Cefiderocol did not suppress cytokine release in human whole blood. In this study, *E. coli* LPS elicited a stronger immune response than *P. aeruginosa* LPS.

**Supplementary Information:**

The online version contains supplementary material available at 10.1007/s00430-026-00883-1.

## Introduction

Sepsis remains a major global health concern and a leading cause of morbidity and mortality worldwide [1]. Sepsis results from a dysregulated immune response to infection, leading to inflammation and organ dysfunction. An important mediator of this process is lipopolysaccharide (LPS), also known as endotoxin, a component found on the outer membrane of Gram-negative bacteria. LPS is a potent immunostimulatory component and can be recognized by the innate immune system by binding to Toll-like receptor 4 (TLR-4) through interaction with other molecules like MD-2, CD14 and LPS binding protein [2–4]. Consequently, downstream signaling pathways are activated resulting in the release of pro-inflammatory cytokines. Because of its immune-stimulating properties, LPS is used both in vitro and in vivo (i.e., the human endotoxin challenge model) to study inflammatory responses [5]. *Escherichia coli*-derived LPS is most commonly used in experimental study, although LPS from other Gram-negative pathogens is available and may elicit divergent immune responses. Timely administered and effective antimicrobial therapy is essential for improving outcomes in sepsis [6, 7]. Among the newer therapeutic options is cefiderocol, a siderophore cephalosporin, that has been developed to target multi-drug resistant Gram-negative bacteria with limited treatment options. Cefiderocol exhibits high affinity for iron and exploits bacterial iron transport systems to enter the cell. Once inside the periplasmic space, cefiderocol binds to penicillin-binding proteins, disrupting cell wall biosynthesis and ultimately leading to bacterial cell death [8]. Beyond their direct antimicrobial activity, it has been observed that some antimicrobial substances have additional immunomodulatory effects [9]. Such effects, including modulation of cytokine production, have been shown for several antibiotics, such as clarithromycin [10], tetracyclines [11] and fosfomycin [12], whereas data for beta-lactams remains limited [9]. Some evidence suggests that cefazolin may influence cytokine production [13]. Recent studies demonstrated that cefiderocol may attenuate cytokine release. [14, 15]. However, these studies used very high LPS concentrations and a murine macrophage model in one study [14] and purified peripheral blood mononuclear cells (PBMCs) in the other [15]. To extent these findings to a more clinically relevant context, we aimed to investigate both the stimulation potential of *Escherichia coli (E. coli)* versus *Pseudomonas aeruginosa (P. aeruginosa)* LPS and the effect of cefiderocol on LPS-induced cytokine responses in human whole blood ex vivo.

## Methods

### Study design and participants

This ex vivo immunological study aimed to evaluate the immune-stimulatory potential of *E. coli* versus *P. aeruginosa* LPS and to compare the human immune response after LPS stimulation with and without cefiderocol addition. Ten healthy volunteers between the age of 18–45 years were included in the study. Main exclusion criteria were use of any medication within one week prior to study day, vaccination within four weeks, clinically relevant illness within four weeks, alcohol abuse, smoking of more than five cigarettes per day, and pregnancy. Baseline characteristics and vital parameters were recorded for all participants. A single blood sample was obtained from each participant by venipuncture in standardized manner. A total of 72 mL of venous blood was drawn into lithium heparinized tubes for the experiment and a routine laboratory assessment including inflammatory markers was performed. No additional procedures or follow-up assessments were conducted beyond this visit.

Written informed consent was obtained from all participants before enrolment in the study. The study protocol was approved by the Ethics Committee of the Medical University of Vienna (EK 1084/2024) and was conducted in accordance with the International Council for Harmonisation Good Clinical Practice guidelines.

### Laboratory procedures

#### LPS and investigational product

*E. coli* LPS and *P. aeruginosa* LPS were added to whole blood at a final concentration of 50 pg/mL, a level comparable to concentrations reported in patients with sepsis [16]. For *E.coli* LPS, the National Institutes of Health (NIH), USA reference endotoxin derived from *E.coli* O113:H10 was used. It was produced in the United States using hot phenol extraction and subsequent purification steps including alcohol precipitation, enzymatic treatment, ultracentrifugation and lyophilization. The endotoxin underwent biological standardization and quality control testing [17]. For *P.aeruginosa* LPS, research-grade lipopolysaccharide from P.aeruginosa serotype 10 (Sigma Aldrich, USA) was used. It was purified by gel-filtration chromatography and supplied as a lyophilized powder preparation with a protein contamination of less than 3% [18]. Cefiderocol (Fetcroja, Shionogi B.V., Netherlands) was added at 100 µg/mL to one experimental group, corresponding to the maximum concentration (c_max_) observed in humans after administration of 2 g cefiderocol [19]. An equivalent volume of 0.9% saline served as control.

#### Ex vivo LPS stimulation

The experiment is depicted in Figure S1. The experiment consisted of two parallel conditions: blood incubated in the presence of cefiderocol and blood incubated without antibiotic. Prior to LPS stimulation, samples in the cefiderocol arm were spiked with cefiderocol, whereas saline was added to the control arm. Following collection, baseline plasma samples were obtained immediately by centrifugation and stored at  − 80 °C. For the LPS stimulation, aliquots were incubated for 4 h at 37 °C under continuous gentle agitation. LPS (either *E. coli* LPS or *P. aeruginosa* LPS) was added either at the start of incubation (4 h condition) or after 2 h of incubation (2 h condition). Moreover, negative controls without added LPS were performed. Consequently, the samples were incubated for 4 h, with LPS exposure lasting 4 h, 2 h, or none. At the end of the respective incubation period, samples were centrifuged (30 min, 500×*g*, 4 °C), and supernatant was stored at  − 80 °C until analysis.

#### Cytokine quantification

The following cytokines were measured in duplicates using a Luminex 7-plex assay (Luminex Performance Assay, R&D Systems, USA) because they are known to be reliably released following LPS stimulation in humans in vivo and ex vivo [20, 21]: Tumour necrosis factor- α (TNF-α), interleukin-1β (IL-1β), interleukin-6 (IL-6), interleukin-8 (IL-8), interleukin-10 (IL-10), interferon-α (IFN-α) and interferon-γ (IFN-γ).

### Statistical analysis

The primary endpoint was the difference in cytokine concentrations after LPS stimulation in the absence or presence of cefiderocol. An additional endpoint was the comparison of cytokine responses between *E. coli* and *P. aeruginosa* LPS. Baseline data is presented as median with interquartile range (IQR) for continuous variables and number (percentage) for categorical variables. Cytokine data were summarized descriptively as mean ± standard deviation or standard error. Cytokine responses were further quantified as the area under the curve (AUC₀–₄) from baseline to 4 h, calculated using the trapezoidal rule. Paired comparisons (cefiderocol-spiked vs. cefiderocol-free samples, *E. coli* vs. *P. aeruginosa* LPS) were analysed using the paired t-test. We calculated percent differences with 95% confidence intervals to express the relative difference in group comparisons. A two-sided p-value < 0.05 was considered statistically significant. Values below the Lower Limit of Quantification were substituted for 0. Statistical analyses and graphical presentations were performed using GraphPad Prism (GraphPad Software, Boston, MA, USA).

### Role of the funding source

This study was funded by Shionogi. The sponsor had no impact on data analysis or interpretation.

## Results

### Participant characteristics

A total of 10 participants were included in the analysis, with baseline characteristics summarized in Table 1. The median age (interquartile range [IQR]) was 27 years (IQR 25–34) and 8 of the 10 participants were female. The median body temperature was within the normal range at 36.4 °C (IQR 36.0–36.5). Routine laboratory assessment indicated no evidence of systemic inflammation with a median leukocyte count of 5.5 G/L (IQR 5.0–6.8) and a low mean CRP concentration of 0.05 mg/dL (IQR 0.03–0.08).

**Table 1:** Participant characteristics

Variable	Reported as		Value
n			10
Age	median (IQR)		27 (25–34)
Sex	n (%)	Male	2 (20.0)
		Female	8 (80.0)
Body temperature (°C)	median (IQR)		36.35 (36.00–36.50)
Total leukocyte count (G/L)	median (IQR)		5.45 (5.04–6.79)
Total neutrophile count (G/L)	median (IQR)		2.80 (2.50–3.83)
CRP (mg/dL)	median (IQR)		0.05 (0.03–0.08)

### Cytokine levels

**Fig. 1:**
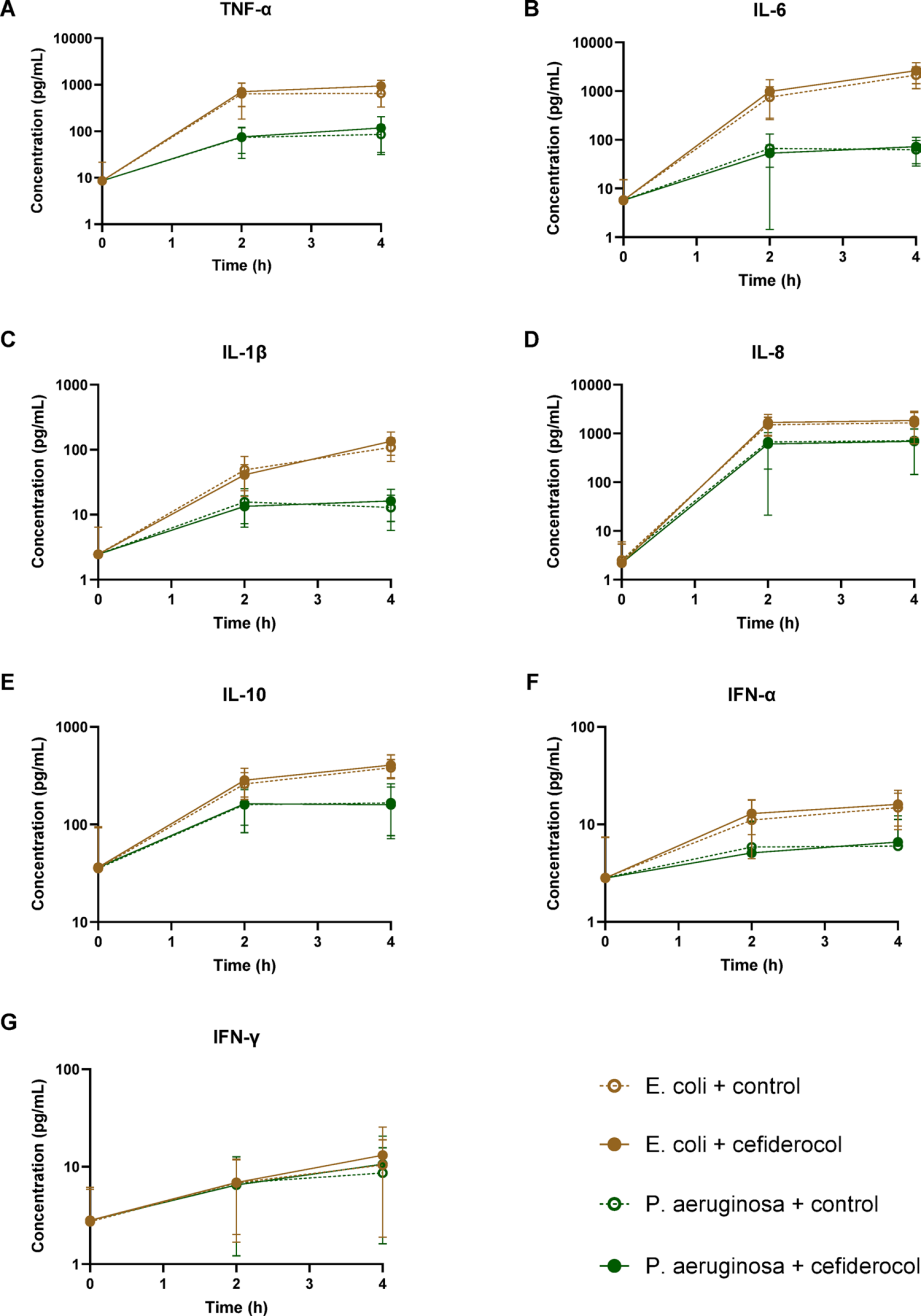
Time-concentration curves of TNF-α, IL-6, IL-1β, IL-8, IL-10, IFN-α and IFN-γ. Data is shown as mean ± standard deviation

**Fig. 2:**
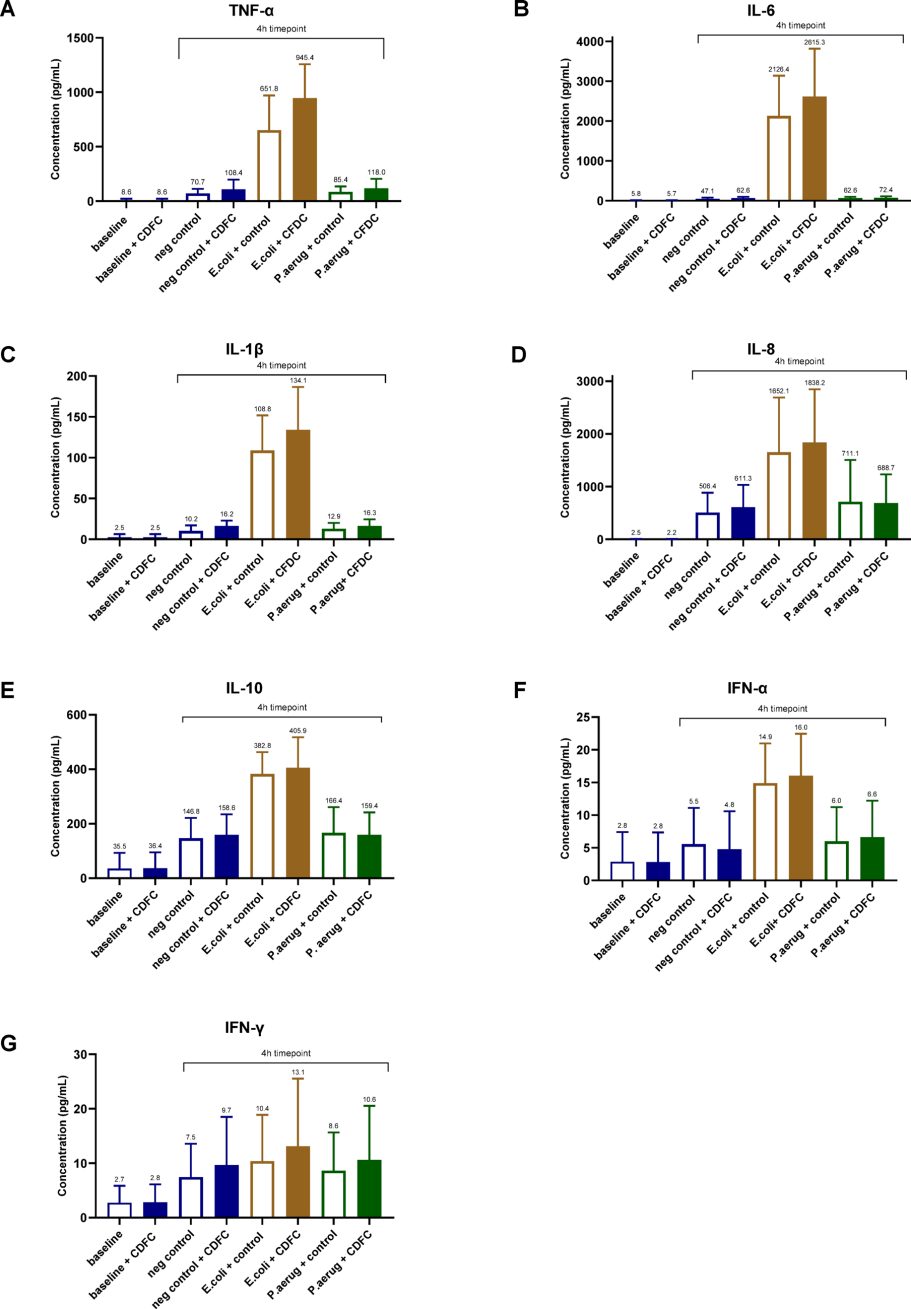
Mean concentration of TNF-α, IL-6, IL-1β, IL-8, IL-10, IFN-α and IFN-γ at baseline and at 4h. Data is shown as mean (horizontal number) ± standard deviation

Cytokine levels were significantly higher after stimulation with *E. coli* LPS compared to *P. aeruginosa* LPS across all tested cytokines. Time-concentration profiles are shown in Fig. 1, and mean concentrations at baseline and 4 h in Fig. 2. Table 2 depicts AUC values with and without cefiderocol and corresponding absolute and percent differences. For TNF-α, the mean (SE) AUC_0-4_ was significantly higher in the presence of cefiderocol than without, both for *E.coli* (2379 ± 613 pg*h/mL vs. 1936 ± 717 pg*h/mL, percent difference 18.2% [95% CI, 5.9–31.4]) and *P. aeruginosa* (278 ± 106 pg*h/mL vs. 242 ± 86 pg*h/mL; percent difference 12.9% [95% CI, 4.3–21.5]). Similarly, for IL-6, cefiderocol increased cytokine release following *E. coli* LPS stimulation (4584 ± 1575 pg*h/mL vs. 3635 ± 1213 pg*h/mL; percent difference 20.7% [95% CI, 8.4–33.0]), whereas no significant difference was observed with *P. aeruginosa* LPS (184 ± 55 pg*h/mL vs. 201 ± 98 pg*h/mL; percent difference -9.3% [95% CI, -53.7–35.2]). For IL-1β, AUCs were comparable with and without cefiderocol for both *E.coli* (219 ± 58 pg*h/mL vs. 209 ± 60 pg*h/mL; percent difference 4.3% [95% CI, 23.1–31.7]) and *P. aeruginosa* (46 ± 13 pg*h/mL vs. 47 ± 15 pg*h/mL; percent difference -2.8% [95% CI, -32.6–27.0]).

**Table 2:** Cytokine AUC_0-4_ values (pg*h/mL) with vs without cefiderocol and corresponding differences

Cytokine	AUC with CDFC	AUC w/o CDFC	Absolute Difference			Percent difference			p value
	mean (SE)	mean (SE)	mean	95% CI		mean	95% CI		
*E. coli*									
TNF-α	2379.40 (613.2)	1935.80 (716.90)	433.50	140.50	746.60	18.22	5.90	31.38	0.01
IL-6	4584.00 (1575.00)	3635.00 (1213.00)	948.60	382.70	1515.00	20.69	8.35	33.05	0.00
IL-1β	218.60 (57.96)	209.20 (60.44)	9.45	-50.39	69.29	4.32	-23.05	31.70	0.73
IL-8	5205.00 (1478.00)	4677.00 (1368.00)	528.30	152.30	904.30	10.15	2.93	17.37	0.01
IL-10	1012.00 (182.80)	939.20 (150.30)	72.43	-36.82	181.70	7.16	-3.64	17.95	0.17
IFN-α	44.61 (10.59)	39.88 (12.06)	4.73	-0.18	9.64	10.60	-0.40	21.61	0.06
IFN-γ	29.65 (14.59)	26.76 (11.61)	2.89	-3.45	9.24	9.76	-11.65	31.16	0.33
*P. aeruginosa*									
TNF-α	278.20 (105.90)	242.2 (85.61)	35.91	11.90	59.93	12.91	4.28	21.54	0.01
IL-6	184.00 (54.79)	201.1 (98.18)	-17.03	-98.87	64.82	-9.26	-53.73	35.23	0.65
IL-1β	45.54 (12.69)	46.81 (15.46)	-1.27	-14.84	12.30	-2.78	-32.59	27.01	0.84
IL-8	1914.00 (812.10)	2048.00 (1211.00)	-133.40	-1021.00	754.60	-6.97	-53.34	39.43	0.74
IL-10	521.60 (136.30)	521.70 (155.70)	0.04	-59.85	59.93	0.01	-11.47	11.49	1.00
IFN-α	19.62 (10.50)	20.54 (9.88)	-0.92	-4.10	2.25	-4.71	-20.91	11.48	0.53
IFN-γ	26.38 (12.86)	24.96 (11.27)	1.42	-3.15	6.00	5.40	-11.94	22.74	0.50

AUC Area-under-the-curve; CDFC cefiderocol; w/o without; p < 0.05 was considered statistically significant.

Among IL-10, IFN-α, IFN-γ and IL-8, there were no significant differences in cytokine levels between groups except for IL-8 where a significant increase in AUC following *E. coli* LPS stimulation in the presence of cefiderocol was observed (percent difference 10.1% [95% CI, 2.9–17.4]).

## Discussion

This ex vivo immunological study investigated two aspects: (i) differences in cytokine production following stimulation with *E. coli* LPS versus *P. aeruginosa* LPS, and (ii) the effect of cefiderocol on LPS-induced cytokine levels in human whole blood. Stimulation with *E. coli* LPS elicited a markedly stronger immune response compared to *P. aeruginosa* LPS, resulting in significantly higher cytokine levels across analytes. In contrast, *P. aeruginosa* LPS did not have a substantial stimulation potential when compared to the 4 h negative control indicating limited immunostimulatory capacity. Cefiderocol did not exhibit anti-inflammatory effects in human whole blood. Cytokine levels were generally higher in cefiderocol-treated samples than in native samples, however, these differences were small and unlikely to be clinically relevant.

Other studies have also demonstrated significantly lower cytokine levels following ex vivo stimulation of cells with *P. aeruginosa* LPS compared to *E.coli* LPS [22, 23]. The observed differences in cytokine release between these species might be attributable to structural variations in the LPS molecule itself. The Lipid A domain, which mediates activation of the innate immune system [4], differs among bacterial species in aspects such as phosphorylation pattern, number of acyl chains or composition of fatty acids [24]. In addition, Lipid A structure may also vary within the same species due to differences in growth conditions, environmental exposure or isolation sources [25]. Such alterations can influence immune recognition and downstream signaling through TLR-4 [2]. The low immunostimulatory activity of *P. aeruginosa* LPS in experimental studies has been attributed to a lower degree of Lipid A acylation [23]. Conversely, LPS derived from *P. aeruginosa* isolates in cystic fibrosis patients exhibits distinct Lipid A modifications, which, however, were associated with an enhanced inflammatory response compared to non cystic fibrosis isolates [26].

Cytokine production following LPS stimulation is complex and influenced by multiple interacting factors including bacterial species, serotype, isolate source, environmental factors and experimental set-up. The NIH *E. coli* reference endotoxin used in this study represents a highly standardized and reproducible LPS preparation that has been widely used for human challenge studies and bioanalytical assays for several decades [27]. The *P. aeruginosa* LPS used in this study represents a purified preparation from a single serotype prepared for research purposes. Therefore, neither preparation can fully reflect the structural diversity of LPS encountered in clinical infections. Furthermore, differences in extraction, purification, and standardization procedures between the two LPS preparations may have influenced LPS structures and thereby contributed to differences in stimulatory activity.

In contrast to our findings previous studies have reported that cefiderocol might attenuate the immune response ex vivo. Hildebrand et al. [15] observed that cefiderocol lead to decreased cytokine production (IL-6, IL-1β and TNF-α) in a dose-dependent manner. However, their experiments used extremely high LPS concentrations (100 ng/ml), which exceeds concentrations typically observed in septic patients approximately 1000-fold [16], and were conducted in isolated peripheral blood mononuclear cells (PBMCs) rather than whole blood. Similarly, Ju et al. [14] reported reduced levels of IL-6, TNF-α, and IL-1β and increased IL-10 levels; however, cultured mouse macrophage cells were used along with excessive LPS levels (100 ng/mL). By contrast, we used freshly drawn human whole blood, which better preserves the complexity of the immune system and used “physiologic” LPS levels observed in human sepsis which may therefore more accurately represent the immune response in vivo.

Our study has some limitations. First, the sample size was small and consisted predominantly of white, young, healthy female participants which may limit generalizability of our findings to other populations. Second, various LPS variants within a species exist and structural heterogeneity between variants may influence Toll-like receptor 4 activation and downstream cytokine release. Third, as this was an ex vivo study, results cannot directly be translated to patients and may not fully reflect the complex host–pathogen interactions occurring in septic patients in vivo. LPS structure may undergo in vivo modifications depending on bacterial strain and host factors, potentially altering immunostimulatory activity. Therefore, extrapolation to clinical settings must be made with caution.

In conclusion, in our study, LPS derived from *E.coli* elicited a robust cytokine response compared to *P. aeruginosa* LPS. Cefiderocol did not demonstrate an immunomodulatory effect in ex vivo in human whole blood.

## Supplementary Information

Below is the link to the electronic supplementary material.

Supplementary Material 1

## Data Availability

Data will be shared upon reasonable request.
