# Supplementary material for: Human ex vivo comparison of Escherichia coli and Pseudomonas aeruginosa lipopolysaccharide–induced immune responses and cefiderocol effects in whole blood
Source: Med Microbiol Immunol. 2026 Jul 27;215(1):20. doi: 10.1007/s00430-026-00883-1 (PMC13407950; doi:10.1007/s00430-026-00883-1)
Supplement: Supplementary file 1 — Supplementary Material 1 [file 430_2026_883_MOESM1_ESM.docx]

**SUPPLEMENTARY APPENDIX**

**Human ex vivo comparison of Escherichia coli and Pseudomonas aeruginosa lipopolysaccharide–induced immune responses and cefiderocol effects in whole blood**

**Figure S1 *Ex vivo* LPS stimulation**
